# Can laypeople identify a drug-induced QT interval prolongation? A psychophysical and eye-tracking experiment examining the ability of nonexperts to interpret an ECG

**DOI:** 10.1093/jamia/ocy183

**Published:** 2019-03-08

**Authors:** Alaa Alahmadi, Alan Davies, Markel Vigo, Caroline Jay

**Affiliations:** University of Manchester, School of Computer Science, Manchester, UK

**Keywords:** drug-induced LQTS, EKG, ECG interpretation, visual perception, patient self-monitoring

## Abstract

**Objective:**

The study sought to quantify a layperson’s ability to detect drug-induced QT interval prolongation on an electrocardiogram (ECG) and determine whether the presentation of the trace affects such detection.

**Materials and Methods:**

Thirty layperson participants took part in a psychophysical and eye-tracking experiment. Following training, participants completed 21 experimental trials, in which each trial consisted of 2 ECGs (a baseline and a comparison stimulus, both with a heart rate of 60 beats/min). The experiment used a 1 alternative forced-choice paradigm, in which participants indicated whether or not they perceived a difference in the QT interval length between the 2 ECGs. The ECG trace was presented in 3 ways: a single complex with the signals aligned by the R wave, a single complex without alignment, and a 10-second rhythm strip. Performance was analyzed using the psychometric function to estimate the just noticeable difference threshold, along with eye-tracking metrics.

**Results:**

The just noticeable difference 50% and 75% thresholds were 30 and 88 ms, respectively, showing that the majority of laypeople were able to detect a clinically significant QT-prolongation at a low normal heart rate. Eye movement data indicated that people were more likely to appraise the rhythm strip stimulus systematically and accurately.

**Conclusions:**

People can quickly be trained to self-monitor, which may help with more rapid identification of drug-induced long QT syndrome and prevent the development of life-threatening complications. The rhythm strip is a better form of presentation than a single complex, as it is less likely to be misinterpreted due to artifacts in the signal.

## BACKGROUND AND SIGNIFICANCE

Drug-induced long QT syndrome (LQTS) is a cardiac abnormality that can increase the risk of a life-threatening arrhythmia, known as torsades de pointes (TdP), which may lead to syncope, drowning, and sudden cardiac death.^1–3^ LQTS is a side effect of more than 100 commonly prescribed QT-prolonging medications including antiarrhythmic drugs, antihistamines, and antidepressants.^4^^,^^5^ People taking these medications may not experience any symptoms, and sometimes a prolonged QT interval can only be detected by examining an electrocardiogram (ECG).^6–8^

An ECG is a graphical representation of the electrical activity of the heart and is widely applied in clinical practice to assess heart function and detect cardiac pathologies.^9^ The QT interval represents the duration of time to complete the ventricular depolarization and repolarization cycle and is measured in the ECG from the beginning of the QRS complex to the end of the T wave.^1^^,^^10^ LQTS occurs when the repolarization of the heart following a heartbeat is delayed and appears as an elongated QT interval on the ECG.^1^^,^^10^ There is also a congenital LQTS caused by mutations in certain genes. People with this disorder might be excluded from using QT-prolonging drugs.^8^^,^^11^

Frequent monitoring is advisable for people who are at high risk of acquiring LQTS including patients who take prescribed QT-prolonging medications^12^ or patients participating in a clinical trial for a new drug.^13^^,^^14^ Several studies have investigated the effectiveness of utilizing ambulatory ECG devices to monitor patients’ ECG remotely,^15^^,^^16^ but this approach still relies on clinicians being able to access and interpret the ECG. An additional complication is that health status, age, sex, and ethnicity all influence a patient’s ECG in general and the QT interval specifically.^10^^,^^17^^,^^18^ It has been shown that there is no “cutoff” value for deciding whether, in isolation, the QT interval is normal, short, or prolonged.^10^ A personalized monitoring solution that considers a patient’s reading against their “normal” baseline ECG has the potential to address some of these issues.

While there are computerized methods for measuring QT interval, the reliability of these methods is limited,^19–26^ and human visual validation is strongly recommended.^20^^,^^23^^,^^27^ In addition to this, the accuracy of automated ECG interpretation methods is affected by several factors including the presence of abnormal sinus rhythm such as atrial arrhythmias^24^ or a poor-quality ECG signal.^23^^,^^24^^,^^27^ Moreover, abstracting the ECG data purely into numbers also risks masking other potential abnormal clinically significant changes in the ECG morphology. For instance, specific T-wave patterns can aid detection of LQTS,^28^ and large T-U waves are known to precede TdP.^29^ As such the ECG morphology still provides the richest information for recognizing LQTS.

Studies have shown that clinicians find QT-prolongation detection difficult.^30^ While QT experts achieve a high level of accuracy (96%), other clinicians, even those who routinely read ECGs, can perform poorly (<25%).^31^ Training is important; in a study in which students were taught to use the tangent method, they performed significantly better than arrhythmia experts and cardiologists.^32^

If patients or their carers or family members can use a clinically reliable ECG monitoring device at home and receive the right training to detect specific types of abnormality, this raises the possibility of self-monitoring outside of the clinical environment. Self-care and self-monitoring have been shown to empower patients with knowledge about their condition, which can reduce anxiety.^33^

Psychophysical experiments are used to model a human’s ability to distinguish a difference in physical stimuli.^34^^,^^35^ In a classical psychophysical experiment, the parameter of interest is typically the difference threshold, which estimates the smallest unit or change of a stimulus a person can detect.^35^

In cardiology, eye-tracking research has been used to study the visual behavior of medical practitioners reading an ECG.^36–38^ To date, studies have neither applied psychophysical methods to understand ECG interpretation nor investigated the ability of laypeople to perceive differences in ECG morphology.

### Objective

The primary objective was to quantify a layperson’s ability to detect a clinically significant drug-induced QT interval prolongation when compared to a “normal” ECG (baseline). The secondary objective was to determine whether the presentation of the ECG (as a single complex or a 10-second rhythm strip) affects this ability.

## MATERIALS AND METHODS

### Participants

Thirty participants (15 men and 15 women) with no experience in ECG interpretation were recruited from a university campus (26 students and 4 staff). The mean age was 26 ± 6 years. Participants were asked to rate their knowledge of ECGs or ECG interpretation; only people who identified as having no knowledge were included.

### Stimuli design

The ECG stimuli were taken from a clinical study conducted to assess QT interval changes in healthy subjects receiving medication known to cause QT prolongation.^39^ As the study is motivated by the potential for self-monitoring, we selected data from a single participant, whose QT interval was seen to rise to prognostically dangerous levels. The subject (a 35-year-old man) had normal QT intervals (QT interval < 430 ms) prior to taking the medication dofetilide (a class III antiarrhythmic); he subsequently experienced a gradual increase in the QT interval, and eventually reached very high QT prolongation (QT interval = 579 ms). The ECGs sampled all had a heart rate of 60 beats/min to ensure it was possible to compare QT intervals without having to apply a heart rate correction formula (QTc). The QT values used were 417, 421, 430, 441, 485, 537, and 579 ms. It was not possible to select a fixed increase of QT interval for 2 reasons. First, the subject experienced a variable increase in the QT interval over 24 hours, after receiving a single dose of the medication. Second, as we limited our selection to ECGs that have a heart rate of 60 beats/min, only 7 ECGs were available for this representative case. The dataset and its sources can be found in the PhysioNet database,^40^ and the clinical trial study can be found in Johannesen et al.^39^

### Study design

The experiment used a counterbalanced within-subjects design with 2 independent variables:

- QT interval difference (see Table 1

**Table 1.** QT values acquired from the clinical trial between the baseline and the comparison stimuli

Trial	Level of Difference	QT Value of the Baseline ECG (ms)	QT Value of the Comparison ECG (ms)	Value of QT Increase	Clinical Rating
1	0 (no difference)	417	417	0	Normal
2	1 (smallest difference)	417	421	4	Normal
3	2	417	430	13	Borderline
4	3	417	441	24	Borderline
5	4	417	485	68	Prolonged
6	5	417	537	120	Very prolonged
7	6 (highest difference)	417	579	162	Very prolonged

*Note*: As the heart rate was 60 beats/min, the QT is the same as the corrected QT (interval using Bazett’s formula. The clinical rating was determined based on the suggested Bazett-corrected QT interval values for diagnosing QT prolongation in adult men.^10^^,^^41^

**Figure 1.**
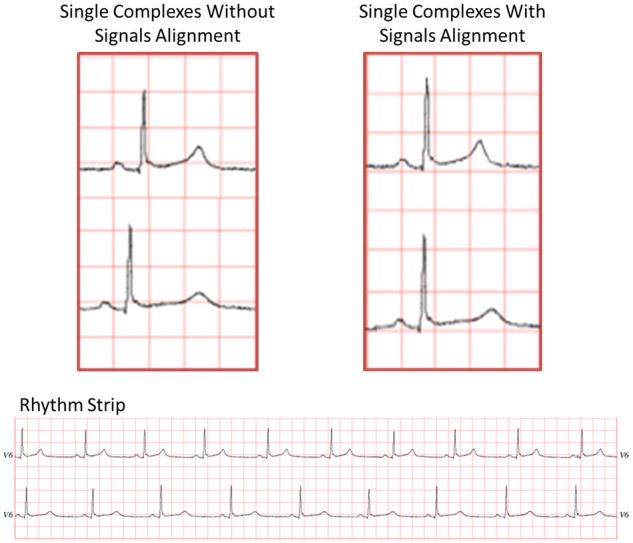
Example of the 3 presentation formats showing a baseline stimulus of a normal QT interval (QT interval = 417 ms, heart rate = 60 beats/min) above a comparison stimulus of a prolonged QT interval (QT interval = 537 ms, heart rate = 60 beats/min).

ECG: electrocardiogram.

), with 7 levels ranging from 0 (no difference) to 6 (highest difference);
- ECG signal presentation format (see Figure 1 ), with 3 versions, in which each consisted of a baseline complex with a normal QT interval and a comparison complex with either a normal or prolonged QT interval:
  1. Two single ECG complexes without R-wave alignment.
  2. Two single ECG complexes aligned on the R wave.
  3. Two 10-second rhythm strips showing 10 complexes.

Each participant completed 21 trials (7 for each presentation format).

We used the method of constant stimuli, in which the levels of QT interval change in the comparison stimulus are presented randomly and are not related from one trial to the next. This reduces errors of habituation and expectation as the participant cannot predict the level of the next stimulus.^42^ Participants completed all trials for one format before moving to the next. The order of presentation formats was counterbalanced across participants.

### Apparatus

A Tobii X2–60 eye tracker and Tobii Studio 3.2 software were used to record eye gaze with a sampling rate of 60 Hz. Gaze coordinates were recorded every 16.7 ms. Audio was recorded to collect participants’ verbal answers.

### Task and procedure

Participants were introduced to the ECG trace and shown how to identify the location of the QT interval. Then, each participant completed an assessment task, in which they were asked to highlight the location of QT intervals on 3 different ECGs. Participants were also shown how to determine the interval length by counting the grid squares between the beginning of the Q wave and the end of the T wave. People were not asked to determine what a normal QT was, but rather to look for a change in its length. Accordingly, this preparation session did not involve any medical terms, clinical methods or high-level training techniques typically associated with ECG interpretation.

The experiment used a classical psychophysical discrimination task known as 1-alternative forced-choice same-different task, also occasionally known as 2IAX or AX.^35^ Participants were presented with 2 ECGs—a baseline stimulus in which the QT interval is normal (no QT interval prolongation) above a comparison stimulus that represents a change in the QT interval—and they had to decide whether the QT intervals of the 2 stimuli were the same or different. We presented the baseline stimulus above the comparison stimulus in all trials, and the participants were aware that the “normal” baseline was always positioned at the top. One trial shows the same ECG for the baseline and the comparison stimuli. Another 6 trials present the baseline as the “normal” QT interval (417 ms), and the comparison as “longer” QT interval of 421, 430, 441, 485, 537, or 579 ms. Table 1 shows the difference between the 2 ECGs in each trial. The participants indicated verbally whether there was a difference in the QT intervals. There was no time limit imposed. The answers were recorded on a paper sheet during the experiment by the researcher and reviewed via the audio recording after the experiment.

### Analysis

Two types of assessment were used to analyze participants’ responses.

#### Assessment 1

For the trial in which the QT interval was the same for the baseline and comparison stimuli (ie, level 0) (Table 1), participants’ responses were assessed for detection of negative findings measured as true negatives (ie, correct reject) and false positives (ie, false alarm). A false alarm response is registered when there is no QT interval difference but participants report that there is, and a correct reject response is recorded when they correctly identify the QT intervals as the same.

#### Assessment 2

For the 6 trials that showed increases in the QT interval (ie, levels 1–6) (Table 1), participants’ responses were assessed for detection of positive findings as true positives (ie, when participants correctly perceived a difference in the QT intervals) and false negatives (ie, when they did not perceive a difference in the QT intervals when a difference was present). This assessment was carried out using the psychometric function, an inferential model applied in psychophysical detection and discrimination tasks. It was used to model the relationship between the gradual increase in the QT interval and the forced-choice responses of the participants. The psychometric function was plotted as the proportion of correct responses as a function of QT interval, and the just noticeable difference (JND) threshold was estimated. In psychophysics, the JND is defined as the minimum amount of change necessary in a stimulus to be just noticeable and detectable.^35^ In this study, we defined it as the minimum amount of QT interval change required to be just discriminable. We estimated the 50% and 75% JND thresholds as the value of QT interval in the comparison stimulus at which the proportion of correct responses is equal to 0.5 and 0.75, respectively. These JND thresholds were then used to determine the point at which participants were able to detect a clinically relevant difference. The equations used for estimating the JND thresholds were defined as follows:

*JND (in ms) = QT value of the comparison stimulus at 50% correct answers – QT value of the baseline stimulus*

**Equation 1.**

The just noticeable difference (50%) threshold estimation formula.

*JND (in ms) = QT value of the comparison stimulus at 75% correct answers – QT value of the baseline stimulus*

**Equation 2.**

The just noticeable difference (75%) threshold estimation formula.

**Figure 2.**
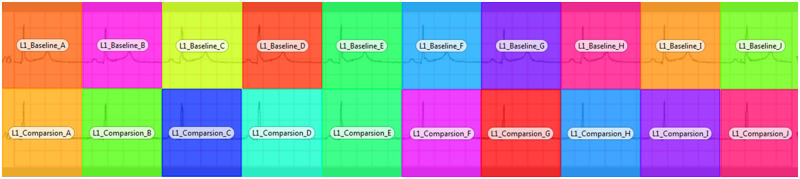
The areas of interest for the rhythm strip presentation format. Each area of interest represents 1 electrocardiogram complex.

To facilitate the calculation of eye movement metrics, areas of interest (AOIs) were created on the stimuli using Tobii studio software. For the single ECG complex presentation format—with or without signals alignment—2 areas of interest were created: 1 for the baseline stimulus and 1 for the comparison stimulus. For the rhythm strip presentation format, an AOI was created for each ECG complex, resulting in 10 AOIs for the baseline stimulus and 10 AOIs for the comparison stimulus. Figure 2 illustrates these areas of interest for the rhythm strip presentation format. The eye-tracking metric total fixation duration, which indicates the total length of time participants fixated on a given AOI, was calculated for the 3 presentation formats (in the case of the rhythm strip stimulus, this was cumulative across all AOIs). Additionally, the percentage fixated metric, which is the percentage of participants who fixated at least once within an AOI, was calculated for each ECG complex in the rhythm strip presentation format.

## RESULTS

### Assessment 1: correct reject and false alarm

For the trial which showed no prolongation of QT interval (ie, the baseline and comparison were the same), the percentage of correct reject responses was 93.33% and false alarm rate was 6.66% in the rhythm strip presentation, demonstrating that only 2 participants of 30 incorrectly perceived a difference in QT interval in which no difference exists. In the case of the single complex without signals alignment, correct reject rate was 90% and false alarm rate was 10%. In the condition with signals alignment, the correct reject rate was 100%.

### Assessment 2: the psychometric function

**Figure 3.**
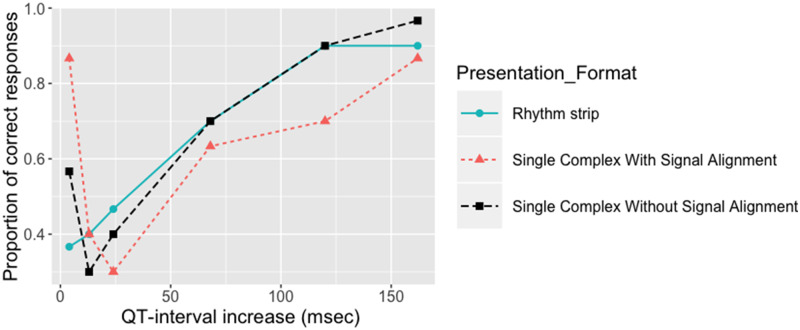
The psychometric function plot showing the proportion of correct responses on the y-axis as a function of QT interval on the x-axis for the 3 presentation formats.

The psychometric function modeling shows an incremental cumulative distribution curve in the rhythm strip presentation, indicating that the proportion of people able to perceive the difference in the QT interval grew as the QT interval increased. Data from the single ECG complex presentations, both with and without signals alignment, showed a different pattern, as a large number of people appeared able to detect the smallest possible difference. As it is unlikely that a person can perceive a small increase in a stimulus level, but not perceive a higher increase, this is likely to be due to an artifact in the particular complex used as a stimulus. Figure 3 illustrates the psychometric function model for the 3 presentation formats.

### JND threshold

The JND was estimated only from the rhythm strip format as it showed the most reliable results. The 50% and 75% JND thresholds were 30 ms (QT interval = 447 ms) and 88 ms (QT interval = 505 ms), respectively, and were determined from fitting the psychometric function using a logistic function with maximum likelihood estimation.

### Total fixation duration

**Figure 4.**
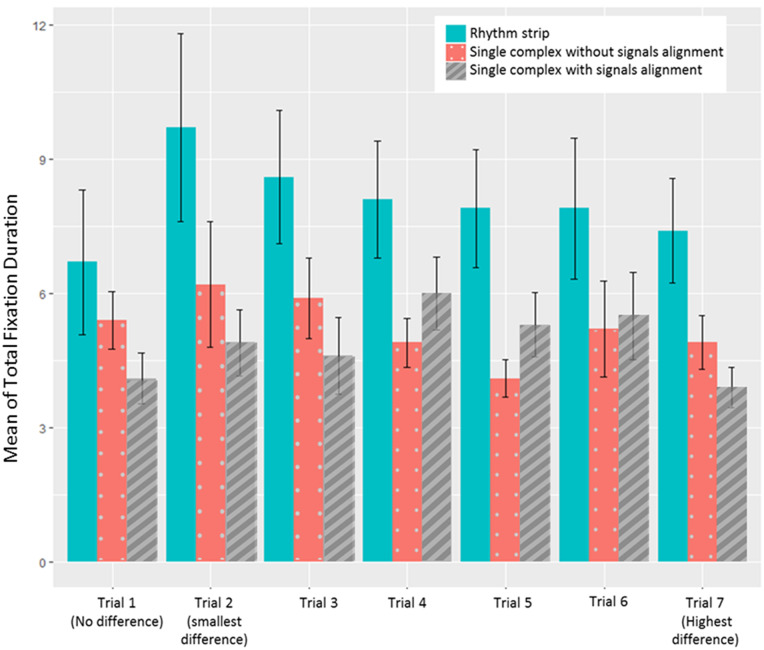
Mean of total fixation duration in seconds for the 3 presentation formats as a function of QT interval difference across all trials. The error bars represent SEM.

The mean of total fixation durations were 3.85 ± 5.21 seconds for the rhythm strip presentation, 1.82 ± 2.21 seconds for the single complex with signal alignment and 1.62 ± 2.75 seconds for the single complex without signal alignment across all trials. The mean of total fixation duration differs significantly between the 3 presentation formats for all trials when compared with a Friedman test, χ^2^(2) = 0.20, *P* < .05, as seen in Figure 4 and Table 2

**Table 2.** Results of the Friedman test comparing the mean of total fixation duration across the 3 presentation formats

Trial	Level of Difference	χ^2^(2)	*P*Value
1	0 (no difference)	7.008	.030
2	1 (smallest difference)	7.681	.021
3	2	7.267	.026
4	3	7.681	.021
5	4	12.067	.002
6	5	7.800	.020
7	6 (highest difference)	14.467	.001

This shows that people fixated significantly longer in the rhythm strip condition than either of the single complex conditions.

### Percentage fixated in the rhythm strip AOIs

**Figure 5.**
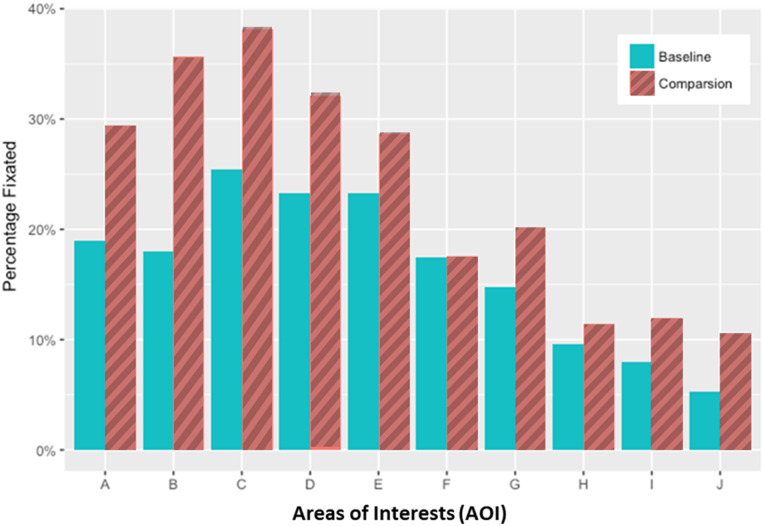
Percentage of people fixating on the areas of interest (AOIs) in the rhythm strip presentation, averaged across all trials. Each AOI represents a single electrocardiogram complex.

The percentage of rhythm strip AOIs fixated was calculated to determine whether people looked at more than 1 ECG complex before making their decision (see Figure 5 ).

**Figure 6.**
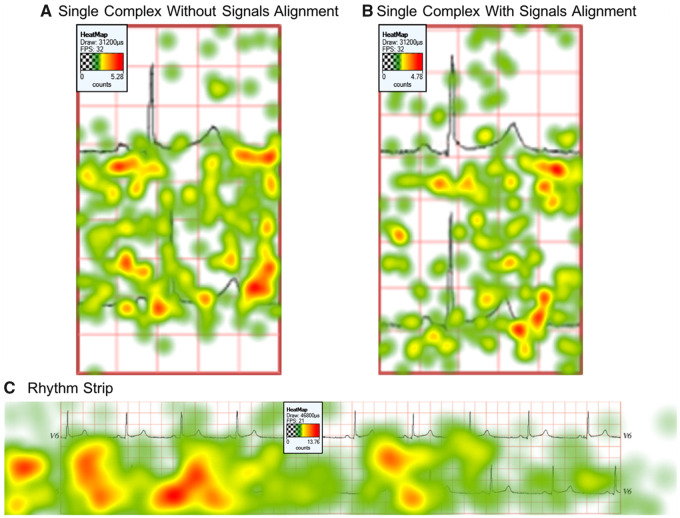
A heatmap of mean fixation count for the 3 presentation formats of trial number 1, showing the smallest difference in QT interval. The presentation formats are (A) single complex without signals alignment, (B) single complex with signals alignment and (C) rhythm strip.

In any given trial, participants fixated on average at least 4 ECG complexes for either the baseline or the comparison stimulus before making their decision. Participants looked at the first 5 ECG complexes (from left to right, ie, the AOIs A–E in Figure 5) more than the other complexes. Heat maps of mean fixation frequency also show this result (Figure 6 c).

## DISCUSSION

The study showed that laypeople can perceive a clinically significant prolongation of the QT interval at a low normal heart rate (60 beats/min) with minimal training. The estimated JND thresholds indicate that 50% of people perceived the difference when the QT interval was borderline (QT interval = 447 ms and JND = 30 ms) and 75% of people perceive an even longer difference (QT interval = 505 ms, JND = 80 ms). This provides evidence that people could be trained to self-monitor for LQTS. Although the QT-prolongation above 500 ms is considered a risk factor for TdP,^43^ clinical research has shown that even a small (∼10 ms) QT interval increase from the baseline is considered a significant side effect of a QT-prolonging drug.^44^^,^^45^

The analysis from both the psychometric function and eye-tracking data show that the rhythm strip presentation is preferable to the single complex presentation, as it is less susceptible to artifacts in the ECG morphology. The psychometric function model showed that participants’ responses in the rhythm strip condition formed a linear curve showing a proportional relationship between the perceived difference and the gradual increase of QT interval. This is in contrast with the single complex presentation, which appeared to show that people were able to detect a very small difference more easily than a longer one. This suggests that people need to view more than 1 ECG complex to come to an accurate decision. The eye-tracking data supports this argument. People looked on average at least 4 ECG complexes before making a decision (Figures 5 and 6C). Figure 6A and 6B show a heatmap of fixations in the single complex presentation, in which the majority occurred on the end of the T wave.

### Study limitations and future work

This study only examined the perception of QT interval prolongation and it is not clear whether laypeople could identify other abnormalities, such as changes in ST-segment elevation. The ECGs had a single, normal heart rate of 60 beats/min. Detecting a difference could be more difficult at higher or lower heart rates, and future work should investigate this. Although detection rates in this study compared favorably to those of some clinicians,^31^ it should be noted that a different paradigm was used in the current study (forced choice rather than classification), and as such the results are not directly comparable. The study examined people’s ability to detect a QT prolongation in terms of sensitivity (identifying true positives). Future work should also examine specificity (identifying true negatives) as well as measuring the predictive positive value, which is the proportion of positive results reported by the participant that are truly positive. This is important for understanding the practical aspects of self-monitoring.

The data used to design the stimuli were acquired from a 12-lead ECG, and not a mobile monitoring device, in which the signal is likely to be less reliable and affected by noise. The psychophysical task employed in this study can yield a biased response, as people may be more inclined to respond by saying “different” or “the same.” A 2-alternative forced-choice task can guard against this, as it forces the participant to choose the stimulus that has the longer QT interval.

## CONCLUSIONS

Laypeople can detect a clinically significant QT interval prolongation in a standard ECG signal presentation, when compared with a “normal” ECG baseline. A rhythm strip, which shows more than 1 ECG complex, is less likely to cause misperception of the QT interval. The results show the potential for training laypeople to self-monitor their ECG outside of the clinical environment, which may help with more rapid identification of drug-induced LQTS, and enable treatment to be altered to prevent the development of life threatening complications.

## FUNDING

This work was supported by the corresponding author’s sponsor Taibah University, Kingdom of Saudi Arabia, College of Computer Science and Engineering, Yanbu (grant number TAU388).

## AUTHOR CONTRIBUTORS

AA and CJ devised the idea for the work. AA designed the study, carried out the data collection, analyzed the results and wrote the paper, with CJ, AD, and MV contributing significant edits. CJ assisted with study design and analysis. AD acted as the electrocardiogram domain expert throughout.
